# Special Issue: “Polymer-Based Biomaterials and Tissue Engineering”

**DOI:** 10.3390/ma16144923

**Published:** 2023-07-10

**Authors:** Roser Sabater i Serra, Ángel Serrano-Aroca

**Affiliations:** 1 Centre for Biomaterials and Tissue Engineering, Universitat Politècnica de València, 46022 Valencia, Spain; 2 Biomedical Research Networking Centre in Bioengineering, Biomaterials and Nanomedicine (CIBER-BBN), 46022 Valencia, Spain; 3 Biomaterials and Bioengineering Lab, Centro de Investigación Traslacional San Alberto Magno, Universidad Católica de Valencia San Vicente Mártir, 46001 Valencia, Spain

Polymers in the form of films, fibers, nano- and microspheres, composites, and porous supports are promising biomaterials for a wide range of advanced biomedical applications: wound healing, controlling drug delivery, anti-cancer therapy, biosensors, stem cell therapy, and tissue engineering. In this regard, polymer-based materials in the form of hydrogels; interpenetrated and semi-interpenetrated polymer networks; composites; or nanocomposites in their pure form, which can be functionalized or used in combination with other materials, nanomaterials, particles, or nanoparticles, can be exploited to produce a broad range of advanced nano- and macro-biomaterials. Specific features, such as mechanical performance, wettability, water diffusion, electroactivity, thermal properties, and even antimicrobial activity, can be tailored to engineer biomimetic microenvironments that are able to promote cellular interactions and tissue development for tissue engineering and regenerative medicine applications while preventing microbial infections. Furthermore, polymers are becoming increasingly important as a primary tool for controlling the release profile of drugs. New approaches have been developed to improve the efficacy of drug therapy, improving healing effectiveness.

This Special Issue presents new approaches in the areas of novel biomaterials for tissue engineering and drug delivery. It includes biomaterials based on polymeric matrices with various structures, such as porous composites, nanofibrous scaffolds, hydrogels, or meshes, which can include micro- or nanoparticles (graphene nanosheets, bioactive glass, and hydroxyapatite and halloysite nanotubes).

Stefaniak and Masek [1] summarized in a short review the copolymerization of the well-known poly(lactic acid) (PLA) with other polymers to improve PLA properties. PLA can be considered an ecological material because it can be produced using renewable resources. The review focuses on PLA copolymerization accomplishments for different applications, especially in pharmaceutical and biomedical fields.

Studies [2,3,4,5] reported novel strategies for the development of biomaterials in the form of composites, hydrogels, or electrospun fibers for tissue engineering applications. Głąb et al. [2] investigated the influence of collagen types on the physico-chemical properties of polyvinylpyrrolidone and poly(vinyl alcohol) composites that also included a ceramic phase (hydroxyapatite). The study demonstrated the great potential of collagen-modified composites for biomedical use, particularly for bone tissue engineering. Aparicio-Collado et al. [3] proposed a novel graphene-based poly(3-hydroxybutyrate-co-3-hydroxyvalerate)/polyvinyl alcohol semi-interpenetrated networks with low amounts of graphene (G) nanosheets within the polymer matrix to produce nanohybrid hydrogels with electroactive properties. The mechanical and electrical properties significantly increased in nanohybrid hydrogels with only 0.2% of G nanosheets, which showed good biocompatibility with muscle cells. The conductive hydrogels, with electrical conductivity in the range of human skeletal muscle tissue, were able to induce myoblast proliferation, indicating its great potential for musculoskeletal tissue engineering. Schuhladen et al. [4] prepared polyhydroxyalkanoate (PHA)/bioactive glass (BG) composites. Bioglass 45S5 (in wt.%: 45.0 SiO_2_, 24.5 Na_2_O, 24.5 CaO, and 6.0 P_2_O_5_) and copper-doped 45S5 BG (in wt.%: 45.0 SiO_2_, 24.5 Na_2_O, 22.0 CaO, 6.0 P_2_O_5_, and 2.5 CuO) were used to engineer PHA/BG porous scaffolds (using the salt-leaching method). Murine stromal ST2 cells cultured in media with different dissolution products released from the scaffolds show a minor reduction in cell viability with an increase in VEFG release, which indicates that the composites are interesting for tissue engineering applications. In addition, Matschegewski et al. [5] prepared electrospun scaffolds from FDA-approved commercial medical-grade polymers (PLA, polycaprolactone (PCL), and polyamide) to assess the physicochemical and biological evaluation of cardiac implants. Untreated and plasma-activated polymeric nonwovens were analyzed to evaluate their influence on endothelial cell response. The study demonstrated the potential of plasma-activated electrospun scaffolds for advanced cardiac implant development.

Studies [6,7,8] focused on different approaches related to drug delivery. Haroosh et at. [6] developed PLA/PCL blends using halloysite nanotubes to obtain a sustained release of hydrophilic drugs. Using tetracycline hydrochloride (TCH) as a drug model, they found that when TCH was loaded into hydrophobic PLA/PCL blends with halloysite nanotubes, drug release decreased, overcoming the weak interaction between TCH and PLA/PCL blends. In another study, Bhanderi et al. [7] reported a novel delivery approach to deliver rivastigmine, a reversible cholinesterase inhibitor, for intranasal applications relative to brain delivery to treat neurodegenerative diseases such as Alzheimer’s disease. The study reports the development of mucoadhesive rivastigmine loaded in chitosan and coated with Eudragit EPO, a cationic terpolymer from the poly(methacrylate) family, for intranasal delivery. This system could circumvent the first-pass metabolism of drugs and help achieve a sustained drug release. The development of microparticles that are able to encapsulate the nanoparticles of compounds for pulmonary drug-delivery applications has been addressed by Sato and Murakami [8]. Temperature-responsive polysaccharide microparticles containing nanoparticles were engineered, which can release two differently charged compounds in a two-step release. The delivery system has great potential to be used as a temperature-responsive drug carrier for various administration routes, such as pulmonary, transpulmonary, intramuscular, and transdermal administration.

Finally, Turlakiewicz et al. [9] focused on the problems related to parastomal hernia and the major properties of surgical meshes (mainly based on polymers but also biological meshes) available on the market. The review also includes the surgical techniques currently used to treat parastomal hernia and post-surgery complications.

We hope that the findings presented in this Special Issue will be useful in ongoing efforts to develop new biomaterials and novel approaches based on polymeric biomaterials for advanced biomedical applications.

## Data Availability

Data sharing is not applicable.
